# The Rice *Basic Helix–Loop–Helix 79* (*OsbHLH079*) Determines Leaf Angle and Grain Shape

**DOI:** 10.3390/ijms21062090

**Published:** 2020-03-18

**Authors:** Hyoseob Seo, Suk-Hwan Kim, Byoung-Doo Lee, Jung-Hyun Lim, Sang-Ji Lee, Gynheung An, Nam-Chon Paek

**Affiliations:** 1 Department of Plant Science, Plant Genomics and Breeding Institute, Research Institute of Agriculture and Life Sciences, Seoul National University, Seoul 08826, Korea; flameseob@snu.ac.kr (H.S.); sukhwan0819@snu.ac.kr (S.-H.K.); bdlee94@snu.ac.kr (B.-D.L.); jh.lim19@cj.net (J.-H.L.); sangjee715@snu.ac.kr (S.-J.L.); 2 Department of Plant Molecular Systems Biotechnology, Crop Biotech Institute, Kyung Hee University, Yongin 17104, Korea; genean@khu.ac.kr

**Keywords:** bHLH transcription factor, lamina joint, leaf angle, long grain, brassinosteroid signaling

## Abstract

Changes in plant architecture, such as leaf size, leaf shape, leaf angle, plant height, and floral organs, have been major factors in improving the yield of cereal crops. Moreover, changes in grain size and weight can also increase yield. Therefore, screens for additional factors affecting plant architecture and grain morphology may enable additional improvements in yield. Among the basic Helix-Loop-Helix (bHLH) transcription factors in rice (*Oryza sativa*), we found an enhancer-trap T-DNA insertion mutant of *OsbHLH079* (termed *osbhlh079-D*). The *osbhlh079-D* mutant showed a wide leaf angle phenotype and produced long grains, similar to the phenotypes of mutants with increased brassinosteroid (BR) levels or enhanced BR signaling. Reverse transcription-quantitative PCR analysis showed that BR signaling-associated genes are largely upregulated in *osbhlh079-D*, but BR biosynthesis-associated genes are not upregulated, compared with its parental *japonica* cultivar ‘Dongjin’. Consistent with this, *osbhlh079-D* was hypersensitive to BR treatment. Scanning electron microscopy revealed that the expansion of cell size in the adaxial side of the lamina joint was responsible for the increase in leaf angle in *osbhlh079-D*. The expression of cell-elongation-associated genes encoding expansins and xyloglucan endotransglycosylases/hydrolases increased in the lamina joints of leaves in *osbhlh079-D*. The regulatory function of OsbHLH079 was further confirmed by analyzing *35S::OsbHLH079* overexpression and *35S::RNAi-OsbHLH079* gene silencing lines. The *35S::OsbHLH079* plants showed similar phenotypes to *osbhlh079-D*, and the *35S::RNAi-OsbHLH079* plants displayed opposite phenotypes to *osbhlh079-D*. Taking these observations together, we propose that *OsbHLH079* functions as a positive regulator of BR signaling in rice.

## 1. Introduction

In cereal crops, leaf angle (defined as the angle between the leaf blade and the leaf sheath) is a key factor determining plant architecture, which also includes plant height, tiller number, and panicle morphology [1,2]. In cereal crops including rice (*Oryza sativa*), plant architecture has been an important agronomic trait for increasing crop yield. In particular, leaf angle is closely associated with photosynthetic capacity [3]. Plants with erect leaves capture more sunlight for photosynthesis and are amenable to much denser planting in populations with a high leaf area index for increasing total grain yield. The lamina joint, which connects the leaf blade and leaf sheath, is central in controlling leaf angle [4], as the degree of leaf inclination largely depends on cell proliferation or cell expansion as well as the cell wall composition at the lamina joint.

Brassinosteroid (BR) phytohormones affect lamina joint morphology and increase leaf angle in rice [5]. BRs are a group of steroid phytohormones that are widely distributed in plants; more than 69 types of BRs have been isolated from diverse plants [6]. BRs play pivotal roles in cell expansion, cell division, vascular bundle differentiation, male fertility, senescence, seed germination, grain filling, photomorphogenesis, flowering time, root growth, and abiotic/biotic stress responses [7,8,9,10,11,12,13]. In rice, BR functions in the regulation of grain size, leaf angle, and yield potential. For instance, several mutants with low BR contents or weak BR signaling, such as *dwarf2* (*d2*), *d11*, and *d61*, exhibit dwarfism and produce short grains and erect leaves [14,15,16]. Additionally, many genes have a role in controlling leaf angle, such as *TILLER ANGLE1* (*Ta1*), *EBISU DWARF* (*D2*), *INCREASED LAMINA INCLINATION1* (*ILI1*), *LEAF INCLINATION2* (*LC2*), *INCREASED LEAF ANGLE1* (*ILA1*), and *SLENDER GRAIN* (*SLG*) [1,17,18,19,20,21]. Moreover, loss-of-function mutants of BR-related genes, including *OsDWARF4* and *OsBRI1*, show improved grain yield due to their ability to be planted at a higher density and their enhanced photosynthetic rate [1,22]. Therefore, understanding the effects of BR on rice architecture has important implications for improving yield.

BR signal transduction has been intensively studied in *Arabidopsis thaliana* [23]. Under normal BR levels, BR interacts with BRASSINOSTEROID INSENSITIVE1 (BRI1) and BRASSINOSTEROID ASSOCIATED RECEPTOR KINASE1 (BAK1), forming a BRI–BR–BAK1 complex [24,25]. This complex inhibits the activity of BRASSINOSTEROID INSENSITIVE2 (BIN2) and activates PHOSPHATASE 2A (PP2A) for the activation of BRASSINAZOLE RESISTANT1 (BZR1). The activated BZR1 is translocated into the nucleus and regulates its downstream genes at the transcriptional level [23,26,27,28].

In rice, the BR signaling pathway remains largely unknown, since only a few components have been reported [23]. BR interacts with OsBRI1 and is involved in the formation of the OsBRI1–OsBAK1 complex [29,30], which inactivates OsBIN2 by an unknown pathway [23]. OsBIN2 phosphorylates OsBZR1, LEAF AND TILLER ANGLE INCREASED CONTROLLER (LIC), and DWARF AND LOW TILLERING (DLT) and inhibits their activities. OsBZR1 upregulates *ILI1* and downregulates *LIC* and *DLT*, thus transmitting the BR signal to their downstream genes, which affect plant growth and development [23].

BR mainly affects cell elongation and cell division; moreover, cell number and cell size largely determine organ size during organogenesis [31,32]. Grain size (GS), another key trait determining yield, is mainly determined by grain length (GL), grain width (GW), and grain thickness, all of which are closely related to cell elongation or cell division. Various genes and quantitative trait loci (QTLs) in rice, such as *GS3*, *GS5*, *GW2*, *GW5*, *GW8*, *GW6a*, *qGL3*, *THOUSAND-GRAIN WEIGHT6* (*TGW6*), and *BIG GRAIN1* (*BG1*), affect grain size by regulating cell number [33,34,35,36,37,38,39,40,41]. In addition, *GS2*/*GL2*, *GL7*, and *POSITIVE REGULATOR OF GRAIN LENGTH1* (*PGL1*) regulate grain size by influencing cell size in rice [40,42,43].

The basic helix–loop–helix (bHLH) domain transcription factors act in various biological processes in animals and plants [44]. In flowering plants, 162 bHLH proteins have been identified in *Arabidopsis thaliana* and 167 in rice [45]. These proteins are divided into two groups: typical bHLH proteins harboring both motifs (basic and HLH motif) bind to DNA through the basic region, whereas atypical, non-DNA-binding bHLH proteins lacking the basic region require other bHLH proteins to bind to DNA as protein dimers [46]. For example, rice ILI1 is an atypical bHLH protein that interacts with the typical bHLH protein OsIBH1 and represses OsIBH1 function [47]. This antagonistic regulation controls cell length in the lamina joint. Several bHLH transcription factors, such as BRASSINOSTEROID UPREGULATED1 (BU1), *O. sativa* BU1-LIKE1 (OsBUL1), and OsbHLH107, are involved in controlling leaf angle or grain size in rice [47,48,49,50].

In this study, we show that OsbHLH079 acts as a key regulator in determining leaf angle and grain length. *OsbHLH079-*overexpressing lines exhibited exaggerated leaf inclination, with longer cells on the adaxial surface of lamina joint. In addition, *OsbHLH079* is involved in modulating grain shape because the *OsbHLH079-*overexpressing mutant produced long grains. Several molecular genetic approaches showed that the function of OsbHLH079 is closely associated with the BR signaling pathway. This study provides new insight into the roles of OsbHLH079 in determining leaf angle and grain shape.

## 2. Results and Discussion

### 2.1. OsbHLH079 Increases Leaf Angle in Rice

**Figure 1:**
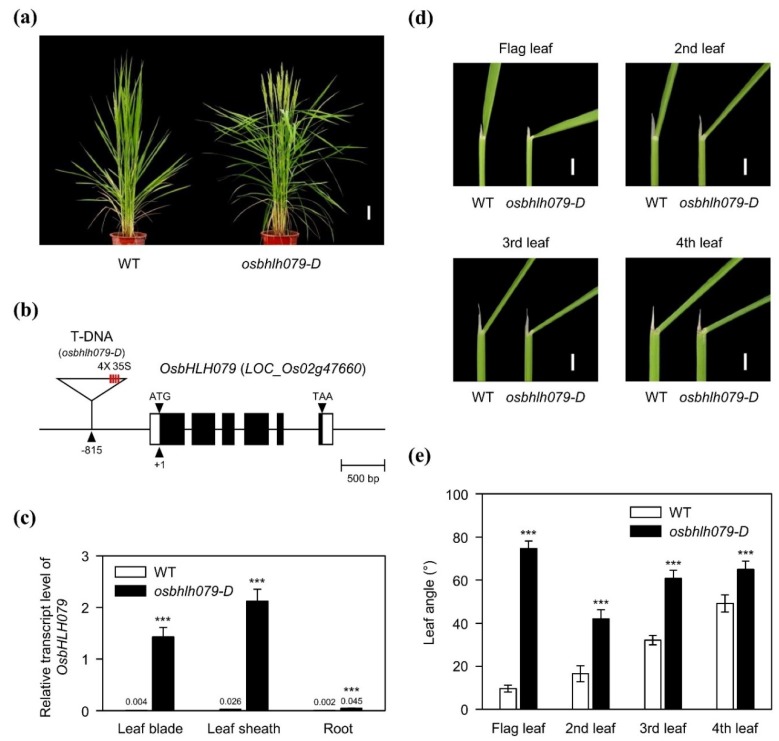
Phenotypic characterization of the *osbhlh079-D* mutant in rice. (**a**) Phenotypes of wild-type (WT) and *osbhlh079-D* at heading stage in plants grown under natural long day (NLD) conditions in the paddy field. Scale bar = 10 cm. (**b**) Schematic diagram illustrating the position of the T-DNA insertion in *OsbHLH079* (LOC_Os02g47660). Open boxes and filled boxes represent the untranslated region and coding sequence of *OsbHLH079*, respectively. (**c**) Comparison of the *OsbHLH079* transcript levels between 3-week-old plants of WT and *osbhlh079-D* grown under natural sunlight in the greenhouse. The transcript level of *OsbHLH079* was measured by RT-qPCR and normalized to *UBQ5*. Means and standard deviations were obtained from five biological replicates. (**d**) The leaf angle phenotypes of WT and *osbhlh079-D* at heading stage grown under NLD conditions in the paddy field. Scale bar = 1 cm. (**e**) Statistical analysis of leaf angles between WT and *osbhlh079-D* at heading stage grown under NLD conditions in the paddy field. Means and standard deviations were obtained from ten biological replicates. Significant differences between means were analyzed using Student’s *t*-test (*** *p* < 0.001). These experiments were repeated twice with similar results.

To identify new components that regulate plant architecture, we screened a collection of T-DNA insertion lines in rice in the Rice Functional Genomic Express Database [51]. We isolated a new mutant with increased leaf angle phenotype (Figure 1a), and found that an enhancer-trap line, PFG_3A-01275, which is derived from the Korean *japonica* rice cultivar ‘Dongjin (hereafter wild type, WT)’ harbors a T-DNA containing four tandem repeats of the CaMV 35S promoter in the promoter of *OsbHLH079* (LOC_Os02g47660) (Figure 1b). To check whether the T-DNA insertion alters the expression of *OsbHLH079*, we compared *OsbHLH079* transcript levels in various organs between WT and the enhancer-trap T-DNA insertion line. RT-qPCR analysis revealed that the transcript levels of *OsbHLH079* in the T-DNA line were much higher in the leaf blade, leaf sheath, and root, compared with WT, although the degrees of overexpression varied among tissues (Figure 1c). Thus, the gain-of-function mutant was termed *osbhlh079-D*.

Next, to characterize the leaf angle phenotype of *osbhlh079-D* in more detail, we compared the leaf angles of the top four leaves between WT and *osbhlh079-D* in field-grown plants at heading stage. The leaf angles of all four top leaves in *osbhlh079-D* were significantly enlarged compared to those in WT, especially those of flag leaves (Figure 1d,e). These results indicate that the overexpression of *OsbHLH079* is closely associated with increase in leaf angle in rice.

**Figure 2:**
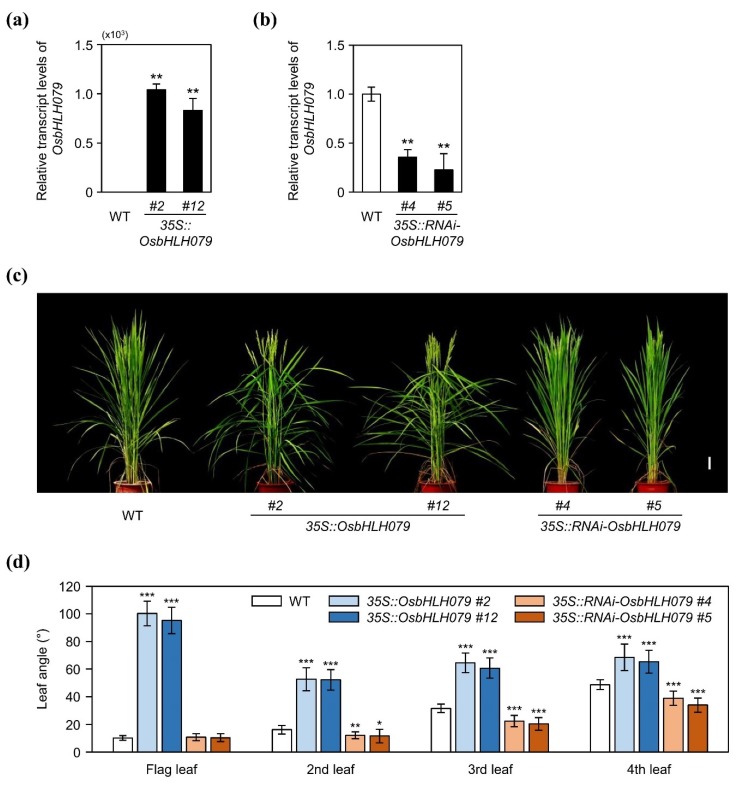
The leaf angles of WT, *35S::OsbHLH079*, and *35S::RNAi-OsbHLH079*. (**a**) Relative transcript levels of *OsbHLH079* in WT, *35S::OsbHLH079 #2*, and *35S::OsbHLH079 #12*. (**b**) Relative transcript levels of *OsbHLH079* in WT, *35S::RNAi-OsbHLH079 #4*, and *35S::RNAi-OsbHLH079 #5*. (**a**,**b**) Total RNA was extracted from the 2-cm lamina joint tissues between the leaf blade and leaf sheath of WT, *35S::OsbHLH079 #2*, *35S::OsbHLH079 #12*, *35S::RNAi-OsbHLH079 #4*, and *35S::RNAi-OsbHLH079 #5* at heading stage in plants grown under NLD conditions in the paddy field. Relative expression levels of *OsbHLH079* were determined by RT-qPCR analysis and normalized to *UBQ5*. Means and standard deviations were obtained from five biological replicates. Differences between means were compared using Student’s *t*-test (** *p* < 0.01). (**c**) Plant phenotypes of WT, *35S::OsbHLH079 #2*, *35S::OsbHLH079 #12*, *35S::RNAi-OsbHLH079 #4*, and *35S::RNAi-OsbHLH079 #5* at heading stage in plants grown under NLD conditions in the paddy field. Scale bar = 10 cm. (**d**) Statistical analysis of leaf angles among WT, *35S::OsbHLH079 #2*, *35S::OsbHLH079 #12*, *35S::RNAi-OsbHLH079 #4*, and *35S::RNAi-OsbHLH079 #5* at heading stage in plants grown under NLD conditions in the paddy field. Means and standard deviations were obtained from ten biological replicates. Significant differences between means were analyzed using Student’s *t*-test (* *p* < 0.05, ** *p* < 0.01, *** *p* < 0.001). These experiments were repeated twice with similar results.

To verify if the overexpression of *OsbHLH079* leads to an increase in leaf angle, we generated two independent transgenic rice lines overexpressing the full-length coding sequence of *OsbHLH079* (*35S::OsbHLH079 #2* and *#12*) as well as two individual RNAi-mediated knockdown lines of *OsbHLH079* (*35S::RNAi-OsbHLH079 #4* and *#5*). First, we checked whether the expression of *OsbHLH079* is altered in the *35S::OsbHLH079* and *35S::RNAi-OsbHLH079* lines. RT-qPCR analysis revealed that the transcript levels of *OsbHLH079* were upregulated in two *35S::OsbHLH079* lines (Figure 2a) and downregulated in two *35S::RNAi-OsbHLH079* lines (Figure 2b). Next, we compared the leaf angles of top four leaves among WT, *35S::OsbHLH079*, and *35S::RNAi-OsbHLH079* at heading stage grown under NLD conditions in the paddy field. Indeed, all the leaf angles of *35S::OsbHLH079* were much larger than WT, especially for the flag leaf, as is the case for *osbhlh079-D*. By contrast, all the leaf angles of *35S::RNAi-OsbHLH079* were significantly smaller, except for the flag leaf angle (Figure 2c,d). Collectively, these results suggested that OsbHLH079 increases leaf angle during leaf blade growth.

### 2.2. OsbHLH079 Increases Grain Length in Rice

**Figure 3:**
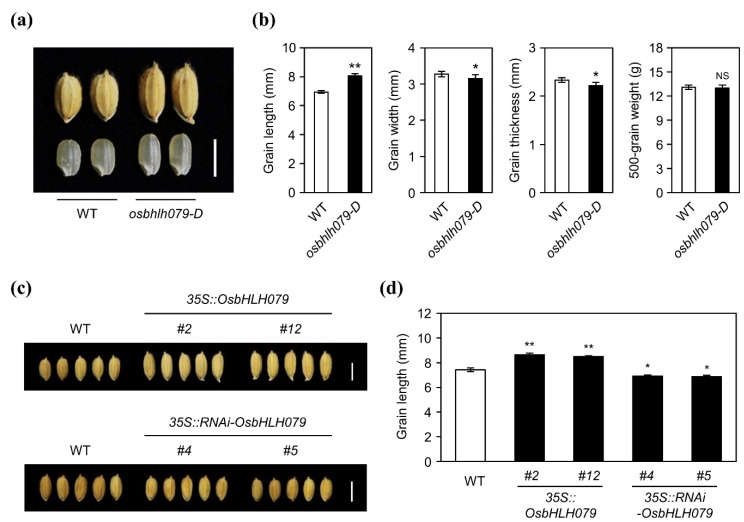
The grain phenotypes of WT, *osbhlh079-D*, *35S::OsbHLH079*, and *35S::RNAi-OsbHLH079*. (**a**) Unhulled and hulled grain phenotypes of the *osbhlh079-D* mutant compared to those of WT. Scale bar = 0.5 cm. (**b**) Comparison of grain length, grain width, grain thickness, and 500-grain weight between WT and the *osbhlh079-D* mutant. Means and standard deviations were obtained from twenty biological replicates. Asterisks indicate statistically significant differences (* *p* < 0.05, ** *p* < 0.01, Student’s *t*-test) compared to WT. NS, not significant. (**c**) The unhulled grain phenotype of *35S::OsbHLH079* (upper panel), and *35S::RNAi-OsbHLH079* (lower panel) compared to that of WT. Scale bar = 0.5 cm. (**d**) Statistical analysis of grain lengths among the WT, *35S::OsbHLH079 #2*, *35S::OsbHLH079 #12*, *35S::RNAi-OsbHLH079 #4*, and *35S::RNAi-OsbHLH079 #5*. Means and standard deviations were obtained from twenty biological replicates. Significant differences between means were analyzed using Student’s *t*-test (* *p* < 0.05, ** *p* < 0.01). These experiments were repeated twice with similar results.

In addition to their increased leaf angle, *osbhlh079-D* plants produced long grains (Figure 3a). The grain length of *osbhlh079-D* was longer than WT, while the grain width and grain thickness of *osbhlh079-D* were smaller, resulting in no significant difference in 500-grain weight between WT and *osbhlh079-D* (Figure 3b). To confirm if the long grain phenotype of *osbhlh079-D* is caused by the overexpression of *OsbHLH079*, we compared the grain length among WT, *35S::OsbHLH079*, and *35S::RNAi-OsbHLH079*. The grain lengths of two independent *35S::OsbHLH079* lines were much longer than WT, whereas the grain lengths of two independent *35S::RNAi-OsbHLH079* lines were significantly shorter than WT (Figure 3c,d). Collectively, these results suggested that *OsbHLH079* is also involved in the regulation of grain length in rice.

### 2.3. OsbHLH079 is a Transcription Factor of the Basic Helix-Loop-Helix (bHLH) Family in Rice

**Figure 4:**
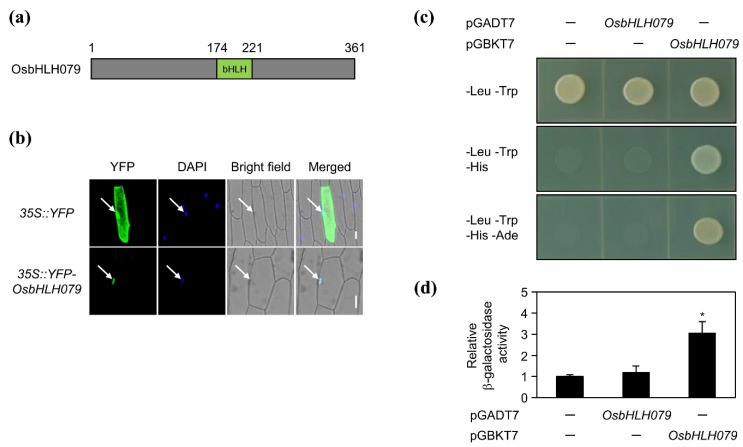
OsbHLH079 as a putative transcription factor. (**a**) Domain analysis of the 361-amino-acid-long OsbHLH079 protein. The green box indicates a basic helix–loop–helix domain. (**b**) Subcellular localization of OsbHLH079 in onion epidermal cells. The *35S::YFP* and *35S::YFP-OsbHLH079* constructs were introduced into onion epidermal cells and the cells were analyzed by confocal laser scanning microscopy at 18 h after particle bombardment. Onion nuclei were stained with DAPI. Arrows indicate the nucleus. Scale bar = 50 μm. DAPI, 4′,6-diamidino-2-phenylindole. (**c**,**d**) Transactivation activity assay of OsbHLH079. The full-length cDNA of *OsbHLH079* was fused with the yeast GAL4 activation domain in the pGADT7 vector, or with the yeast GAL4 DNA-binding domain in the pGBKT7 vector, and the fusion proteins were expressed in the yeast strain AH109. (**c**) Transformed yeasts were grown on the Leu^–^ Trp^–^, Leu^–^ Trp^–^ His^–^, and Leu^–^ Trp^–^ His^–^ Ade^–^ agar media for yeast cell survival assay. (**d**) LacZ activity was obtained using the β-galactosidase liquid assay. The relative β-galactosidase activity was obtained by normalizing to the activity level of the negative control. Means and standard deviations were obtained from three biological samples. Significant differences between means were analyzed using Student’s *t*-test (* *p* < 0.05). These experiments were repeated twice with similar results. -, empty vector.

The domains of OsbHLH079 were analyzed using the NCBI-BLASTP program [52]. OsbHLH079 has a conserved basic helix–loop–helix (bHLH) domain from the 174th to 221th amino acids (Figure 4a). Moreover, the bHLH domain was found to be a putative G-box binding type, which directly binds to the G-box motif in the rice genome, in a previous genome-wide analysis [45]. These data suggested that OsbHLH079 is a bHLH-type G-box binding transcription factor. To determine if OsbHLH079 acts as a transcription factor, we first examined its subcellular localization in onion epidermal cells. The *35S::YFP* (control) and *35S::YFP-OsbHLH079* constructs were introduced into the onion epidermal cells by particle bombardment, and, at 18 h after particle bombardment, onion nuclei were stained with DAPI to detect the nucleus. Confocal laser scanning microscopy showed that YFP-OsbHLH079 fusion proteins exclusively localized in the DAPI-stained nuclei, while YFP proteins were detected throughout the cells (Figure 4b). Next, we performed a transactivation activity assay for OsbHLH079 in yeast. The full-length cDNA of *OsbHLH079* was fused with the yeast GAL4 activation domain in the pGADT7 vector, or with the yeast GAL4 DNA-binding domain in the pGBKT7 vector. Then, the yeast strain AH109, harboring the *HIS3*, *ADE2*, and *LacZ* reporter genes, was co-transformed with a pair of plasmids and plated on each selective medium, as shown in Figure 4c. Only the yeast expressing GAL4BD-OsbHLH079 grew on the selective medium lacking histidine and adenine (Figure 4c). Furthermore, in the β-galactosidase liquid assay, LacZ activity was highly upregulated in the yeast expressing GAL4BD-OsbHLH079 compared to that in the negative control (Figure 4d), indicating that OsbHLH079 has transactivation activity. Taking these observations together, it can be concluded that OsbHLH079 functions as a transcription factor of the basic helix–loop–helix (bHLH) family in rice.

### 2.4. OsbHLH079 Enlarges Cell Size in the Adaxial Side of Leaf Lamina Joints by Upregulating Cell Expansion-Related Genes

**Figure 5:**
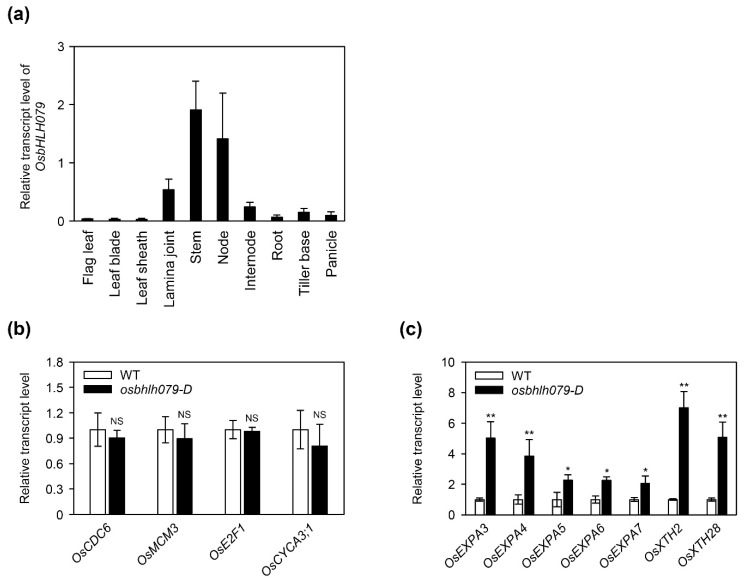
Expression of cell cycle- and cell elongation-related genes in *osbhlh079-D*. (**a**) Spatial expression patterns of *OsbHLH079* in WT at the heading stage grown under NLD conditions in the paddy field. The transcript level of *OsbHLH079* was determined by RT-qPCR analysis and normalized to *UBQ5*. Means and standard deviations were obtained from three biological replicates. (**b**) Expression patterns of cell cycle-related genes in the *osbhlh079-D* mutant compared to those in WT. (**b**) Altered expressions of cell elongation-related genes in the *osbhlh079-D* mutant compared to those in WT. (**b**,**c**) Total RNA was extracted from the 2-cm lamina joint segments between leaf blade and leaf sheath of 2-week-old WT and *osbhlh079-D* grown under long day (LD) conditions (14.5 h light, 30 °C/9.5 h dark, 24 °C) with 60% relative humidity in a growth chamber. The transcript level of each gene was determined by RT-qPCR analysis and normalized to that of *UBQ5*. Means and standard deviations were obtained from three biological replicates Significant differences between means were analyzed using Student’s *t*-test (* *p* < 0.05, ** *p* < 0.01). These experiments were repeated twice with similar results. NS, not significant.

In general, the tissue-specific expression of genes is closely associated with their biological functions. Therefore, we first checked the spatial expression patterns of *OsbHLH079* in field-grown (NLD conditions) WT at heading stage. This revealed that *OsbHLH079* is mainly expressed in the stem, node, internode, and lamina joint (Figure 5a). Previous studies showed that several genes controlling leaf angle are highly expressed in the lamina joint, as is the case of *OsbHLH079*, and the degree of leaf inclination is mainly regulated by cell proliferation and/or cell expansion in the lamina joint, especially in the adaxial side of the lamina joint [5,20,21,41,53,54,55,56,57]. Therefore, we speculated that the expression levels of cell proliferation- or expansion-related genes in lamina joint would be altered in *osbhlh079-D*, and thus compared the transcript levels of those genes in the lamina joint between WT and *osbhlh079-D* by RT-qPCR analysis. The expression levels of cell proliferation-related genes, including *OsCDC6*, *OsMCM3*, *OsE2F1*, and *OsCYCA3;1* [58], in the lamina joint were not significantly different between WT and *osbhlh079-D* (Figure 5b). However, the transcript levels of cell expansion-related genes, such as *OsEXPAs* and *OsXTHs* [59,60], were highly upregulated in the lamina joint of *osbhlh079-D* compared to WT (Figure 5c). Therefore, we hypothesized that the increased leaf angle of *osbhlh079-D* might be caused by expansion of cell size, mainly in the adaxial side of lamina joints.

**Figure 6:**
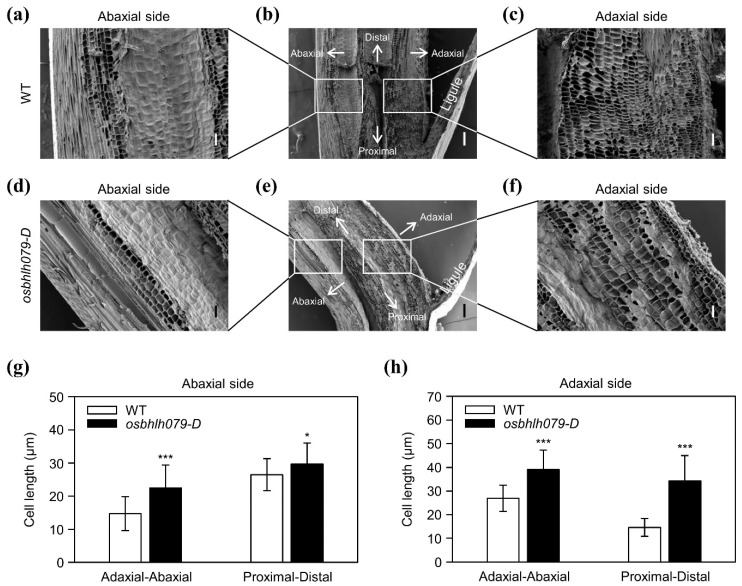
Scanning electron microscopy of the lamina joints of leaves in WT and *osbhlh079-D*. (**a**–**f**) Longitudinal sections of the lamina joint of flag leaf in WT (**a**–**c**) or *osbhlh079-D* (**d**–**f**) at heading stage grown under NLD conditions in the paddy field. (**a**,**d**) Close-up of abaxial regions denoted by rectangles (left side) in (**b**,**e**), respectively. (**c**,**f**) Close-up of adaxial regions denoted by rectangles (right side) in (**b**,**e**), respectively. Scale bar = 200 μm in (**b**,**e**). Scale bar = 50 μm in (**a**,**c**,**d**,**f**). (**g**) Statistical analysis of cell lengths in (**a**,**d**). (**h**) Statistical analysis of cell lengths in (**c**,**f**). (**g**,**h**) Cell lengths along the adaxial–abaxial axis and proximal–distal axis were measured on the abaxial and adaxial sides of lamina joints. Means and standard deviations were obtained from thirty cells. Significant differences between means were analyzed using Student’s *t*-test (* *p* < 0.05, *** *p* < 0.001). These experiments were repeated twice with similar results.

Table 079. *D*, we observed longitudinal sections of flag–leaf lamina joints in WT and *osbhlh079-D* by scanning electron microscopy. The cell length on the adaxial side in *osbhlh079-D* was much larger than WT along the adaxial–abaxial and proximal–distal axes; the abaxial cell size in *osbhlh079-D* was also slightly increased in both axes (Figure 6a–h). These results suggested that OsbHLH079 increases leaf angle by expanding the cell size on the adaxial side of the lamina joint through the upregulation of *OsEXPA* and *OsXTH* genes (Figure 5c).

### 2.5. OsbHLH079 Regulates the Expression of BR Signaling-Related Genes

The wide leaf angle phenotype of *osbhlh079-D* resembles that of mutants with elevated BR accumulation or enhanced BR signaling [11,14,15,61,62,63]. Moreover, the transcript levels of several XTHs and expansin genes, which are upregulated in *osbhlh079-D* (Figure 5c), are significantly increased by BR treatment in *Arabidopsis thaliana*, rice, soybean (*Glycine max*), maize (*Zea mays*), and wheat (*Triticum aestivum*) [59,64,65,66,67,68,69,70]. Therefore, we speculated that the increased leaf angle of *osbhlh079-D* is caused by either elevated endogenous BR accumulation or enhanced BR signaling.

**Figure 7:**
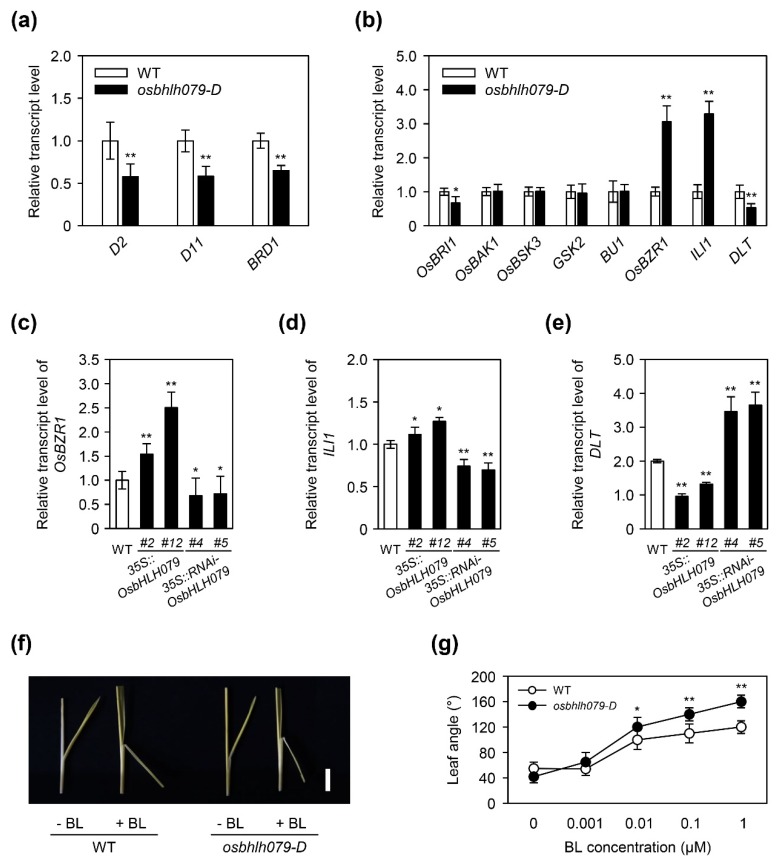
OsbHLH079 acts as a positive regulator of the brassinosteroid signaling pathway. (**a**) Expression patterns of brassinosteroid (BR) biosynthesis-related genes in the *osbhlh079-D* mutant compared to those in WT. (**b**) Altered expressions of BR signaling-related genes in the *osbhlh079-D* mutant compared to those in WT. (**c**–**e**) Altered expressions of *OsBZR1* (**c**), *ILI1* (D), and *DLT* (E) in *35S::OsbHLH079* and *35S::RNAi-OsbHLH079* compared to those in WT. (**a**–**e**) Total RNA was extracted from the 2-cm lamina joints between leaf blade and leaf sheath of 4-week-old plants of WT, *osbhlh079-D*, *35S::OsbHLH079*, and *35S::RNAi-OsbHLH079* grown under LD conditions (14.5 h light, 30 °C/9.5 h dark, 24 °C) with 60% relative humidity in a growth chamber. The transcript level of each gene was determined by RT-qPCR analysis and normalized to *UBQ5*. Means and standard deviations were obtained from three biological replicates. Asterisks indicate statistically significant differences (* *p* < 0.05, ** *p* < 0.01, Student’s *t*-test) compared to WT. (**f**) BR-induced lamina joint inclination in WT and the *osbhlh079-D* mutant. The 2-cm lamina joint segments of 10-day-old seedlings of WT and *osbhlh079-D* grown at 30 °C in darkness were treated with 1 µM BL for 48 h in darkness. Scale bar = 0.5 cm. BL, 24-epibrassinolide. (**g**) Dose-dependent responses of the lamina joint of WT and *osbhlh079-D* to various concentrations of BL. Means and standard deviations were obtained from more than ten biological replicates. Significant differences between means were analyzed using Student’s *t*-test (* *p* < 0.05, ** *p* < 0.01). These experiments were repeated twice with similar results. BL, 24-epibrassinolide.

To investigate whether the expression of BR biosynthesis- or BR signaling-related genes is altered in *osbhlh079-D*, we compared their transcript levels in the lamina joints of leaf blades between WT and *osbhlh079-D*. In the lamina joints, the expression of BR biosynthesis-related genes, such as *D2*, *D11*, and *BRD1* [15,16,61], was significantly downregulated in *osbhlh079-D* compared to that of WT (Figure 7a). In addition, the transcript level of *OsBRI1*, the BR receptor, was also significantly downregulated compared to that of WT (Figure 7b), indicating a negative feedback regulation by enhanced BR signaling [14,63,71]. Among the BR signaling-related genes, including *OsBAK1*, *OsBSK3*, *GSK2*, *BU1*, *OsBZR1*, *ILI1*, and *DLT* [14,30,47,48,72,73,74,75], the expression of *OsBZR1*, and its downstream genes, such as *ILI1*, and *DLT*, was significantly altered in the lamina joint of *osbhlh079-D* compared to WT (Figure 7b). For example, the expression of genes encoding positive regulators of the BR signaling pathway, such as *OsBZR1*, and *ILI1*, was highly upregulated, but the transcript level of *DLT*, which also encodes a positive regulator of BR signaling pathway but is repressed directly by OsBZR1, was significantly downregulated in *osbhlh079-D* (Figure 7b). To confirm whether the expression levels of *OsBZR1*, *ILI1*, and *DLT* are altered by the ectopic or knockdown expression of *OsbHLH079*, we compared the expression levels of *OsBZR1*, *ILI1*, and *DLT* in the lamina joint among WT, *35S::OsbHLH079*, and *35S::RNAi-OsbHLH079*. The transcript levels of *OsBZR1*, and *ILI1* were highly upregulated, while *DLT* expression was significantly downregulated in the lamina joint of *35S::OsbHLH079* lines, as in *osbhlh079-D* (Figure 7c–e). By contrast, the expression levels of *OsBZR1*, and *ILI1* were significantly decreased, while the transcript level of *DLT* was highly increased in the lamina joint of *35S::RNAi-OsbHLH079* lines (Figure 7c–e). These results indicated that the increased activity of OsbHLH079 enhances the BR signaling pathway by altering the expression of *OsBZR1* and its downstream genes, such as *ILI1*, and *DLT*.

**Figure 8:**
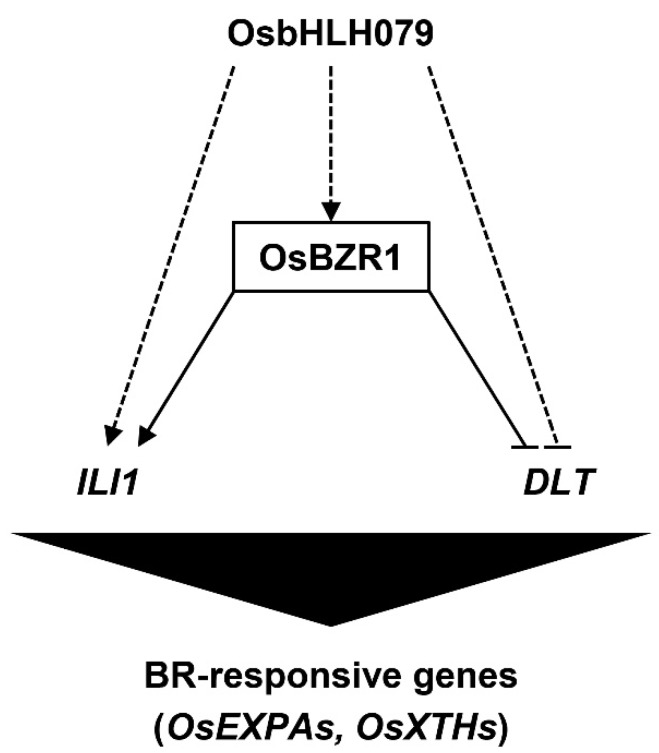
A proposed model of the OsbHLH079-mediated regulatory network in the BR signaling pathway. OsbHLH079 enhances brassinosteroid signaling by upregulating genes encoding positive regulators of the BR signaling pathway, such as *OsBZR1*, and *ILI1*, and downregulating *DLT*, which also encodes a positive regulator of BR signaling and downregulated directly by OsBZR1. Then, altered expressions of BR-responsive genes such as *OsEXPA*, and *OsXTH* produce changes in leaf angle and grain length. Arrows and bars indicate positive and negative regulation, respectively. Solid and dashed lines indicate direct regulation and possible feed-forward regulation, respectively.

To verify whether the response to BR treatment is enhanced by the overexpression of *OsbHLH079*, we carried out a BR-induced lamina joint inclination assay. For this assay, 2-cm lamina joint segments were detached from 10-day-old seedlings of WT and *osbhlh079-D* grown in darkness and treated with 1 µM BL for 48 h in darkness. Then, we compared the extent of lamina inclination of *osbhlh079-D* with WT. As shown in Figure 7f, *osbhlh079-D* was more sensitive to BR (24-epibrassinolide) treatment. Moreover, the difference in the extents of lamina inclination between WT and *osbhlh079-D* increased as the BR concentration increased (Figure 7b). These data indicated that BR signaling is enhanced in *osbhlh079-D*. Therefore, we concluded that OsbHLH079 enhances the BR signaling pathway, which leads to the expansion of cell size in the adaxial side of lamina joints via upregulation of *OsEXPAs* and *OsXTHs*, resulting in an increase in leaf angle in rice (Figure 8).

### 2.6. OsbHLH079 Might Indirectly Regulate Expressions of OsBZR1, and ILI1

As OsBZR1 directly regulates the expression of *ILI1*, and *DLT* [23], it is possible that (1) OsbHLH079 modulates the expression and/or activity of OsBZR1, (2) OsbHLH079 directly regulates other genes because OsBZR1 has a limited function, in which the effect of overexpressed OsBZR1 occurs only when the binding site of negative regulators, such as 14-3-3 or GSK2, is mutated in plants [23,63,76], or (3) OsbHLH079 directly regulates downstream genes of *OsBZR1*, such as *ILI1*, and *DLT*, and expression of *OsBZR1* is increased by a feedback regulatory loop.

To examine the possible roles of OsbHLH079 in the transcription of *OsBZR1*, *ILI1*, and *DLT* in rice, we investigated the promoter sequences of *OsBZR1*, *ILI1*, and *DLT* (−2000 to −1 from the ATG). It revealed that *OsBZR1* or *ILI1* did not contain the G-box sequence (CACGTG; a putative binding site of bHLH-type transcription factors), but only one CACGTG sequence in the promoter region (-989 to −984 from ATG) of *DLT*. These findings suggest that OsbHLH079 regulates *OsBZR1* and *ILI1* indirectly, although we cannot exclude the possibility that OsbHLH079 binds to the promoter regions of *OsBZR1* and *ILI1*.

### 2.7. OsbHLH079 Might Directly Regulate the Cell Expansion-Associated Genes

*PHYTOCHROME INTERACTING FACTOR LIKE1* (*OsPIL1*) functions as a key regulator of internode elongation [77]. *OsPIL1*-overexpressing rice plants (*Ubi::OsPIL1*) formed elongated internodes via larger cells through direct regulation of its downstream genes, such as *OsEXPA4* and *1-ACC OXIDASE*, via binding to the G-box element. Both OsPIL1 and OsbHLH079 are bHLH-type transcription factors. Thus, it can be speculated that OsbHLH079 directly binds to the promoter regions of cell expansion-related genes, such as *OsEXPAs* and *OsXTHs*. The expression of cell expansion-related genes was increased to a much greater extent in the *osbhlh079-D* mutant compared to the increased expression of BR signaling-associated genes (*OsBZR1*, and *ILI1*) (Figure 5c, Figure 7b). The increased expression of BR-related genes in *osbhlh079-D* and *OsbHLH079*-overexpressing plants may have caused a significant change in the expression of the downstream genes, but we could not exclude the possibility that OsbHLH079 directly regulates the cell expansion-associated genes.

### 2.8. OsbHLH079 Increases Grain Length by Altering TGW6 Expression

In rice, several genes and QTLs, including *GS3*, *GS5*, *GW2*, *GW5*, *GW8*, *TGW6*, *GW6a*, *qGL3*, and *BG1*, affect grain size by regulating cell number, and *GS2*/*GL2*, *GL7*, and *PGL1* affect grain size by influencing cell size [33,34,35,36,37,38,39,40,41,42,43]. GW2 is a RING-type E3 ubiquitin ligase and acts as a negative regulator of cell division [33]. *GW2*-overexpressing transgenic rice showed a reduced grain-width phenotype. Increased expression of *GW2* could be one of the reasons for the slender-grain phenotype of the *osbhlh079-D* mutant (Figure S1). *TGW6* encodes an IAA-glucose hydrolase, and loss of function of *TGW6* results in enhanced grain weight, resulting in increased grain yield [38]. *RNAi-TGW6* rice plants showed an increased-grain-length phenotype, which could be one of the reasons for the long-grain phenotype of the *osbhlh079-D* mutant (Figure S1). It is possible that the regulatory function of OsbHLH079 is associated with not only the BR signaling pathway, but also the auxin-related pathway, which is closely involved in the control of grain size and grain yield [38].

### 2.9. OsbHLH079 is an Ortholog of Arabidopsis CRYPTOCHROME-INTERACTING bHLH 1

In the genome-wide analysis, 167 *bHLH* genes in rice and 162 *bHLH* genes in *Arabidopsis* were identified and analyzed by amino acid sequence-based alignments. As a result, they were divided into 25 subfamilies [45]. *OsbHLH079* belongs to the C group with 24 genes in rice and 23 genes in *Arabidopsis*. The C group contains all the *Arabidopsis CRYPTOCHROME-INTERACTING bHLH* (*AtCIB*) genes (*AtCIB1, AtCIB2, AtCIB3, AtCIB4*, and *AtCIB5*), and *OsbHLH079* was annotated to be an *AtCIB1*-like gene [45]. In *Arabidopsis*, CIB1 interacts with cryptochrome 2 (CRY2), and this complex affects various developmental processes, such as hypocotyl elongation, flowering time, stomata opening, hypocotyl bending, programmed cell death, plastid development, and silique elongation [35,78,79,80,81,82,83,84,85]. In soybean, GmCIB1 binds to the E-box (CANNTG) motif and acts as a transcriptional activator to regulate leaf senescence-associated genes [86]. In rice, OsbHLH079 might be an ortholog of CIB1, and the bHLH domain was a highly conserved among amino acid sequences of AtCIB1, GmCIB1, and OsbHLH079 (Figure S2). Additionally, the *osbhlh079-D* mutant flowered earlier than WT under long-day and short-day conditions (Figure S3), which provides more insight into additional regulatory functions of OsbHLH079 in rice growth and development, similar to AtCIB1.

## 3. Materials and Methods

### 3.1. Plant Materials and Growth Conditions

The enhancer-trap T-DNA insertion mutant of *OsbHLH079* (LOC_Os02g47660; PFG_3A-01275; designated as *osbhlh079-D*) in rice was isolated from the Korean *japonica* cultivar ‘Dongjin’, and obtained from the Rice Functional Genomic Express Database [51]. For phenotypic characterization, rice plants were grown under natural long day (NLD) conditions in the paddy field (37°N latitude, Suwon, Republic of Korea). Rice was also grown in growth chambers under long-day (14.5 h light, 30 °C/9.5 h dark, 24 °C) conditions or short-day (10 h light, 30 °C/14 h dark, 24 °C) conditions with 60% relative humidity. The light sources used in the artificial growth chamber were light-emitting diodes (LEDs), and the average photon flux density was around 500 µmol m^−2^ s^−1^.

### 3.2. Vector Construction and Rice Transformation

To generate the *35S::OsbHLH079*, and *35S::RNAi-OsbHLH079* transgenic rice plants, the full-length cDNA of *OsbHLH079*, and the partial cDNA fragment of *OsbHLH079* were amplified from the first-strand cDNA obtained from leaves of WT by reverse-transcription polymerase chain reaction (RT-PCR) using gene-specific primers (Table S1), and subcloned into pCR8/GW/TOPO (Invitrogen, USA). After confirming the sequences, the full-length cDNA of *OsbHLH079*, and the partial cDNA fragment of *OsbHLH079* were transferred into the pMDC32 Gateway binary vector [87], and the pANDA vector [88], respectively, by LR reaction using Gateway LR Clonase II Enzyme Mix (Invitrogen, USA). The resulting constructs, *35S::OsbHLH079*, and *35S::RNAi-OsbHLH079*, were transformed into *Agrobacterium tumefaciens* strain LBA4404, and then introduced into calli generated from mature embryos of WT through *Agrobacterium*-mediated transformation, respectively [89].

### 3.3. RNA Extraction and Reverse Transcription-Quantitative PCR (RT-qPCR) Analysis

Total RNA was extracted from 2-cm lamina joint segments or other tissues using the MG Total RNA Extraction Kit (Macrogen, Seoul, Republic of Korea) according to the manufacturer’s instructions. First-strand cDNAs were synthesized from 2 µg of total RNA using oligo(dT)_15_ primers and M-MLV reverse transcriptase (Promega, USA). The relative transcript levels of each gene were measured by quantitative PCR (qPCR) using gene-specific primers, and rice *Ubiquitin5* (*UBQ5*) was used as an internal control (Table S1) [90]. GoTaq qPCR Master Mix (Promega, USA) was used in a 20 µl total reaction volume, and quantitative PCR was performed using a LightCycler 480 (Roche, Switzerland). qPCR conditions were 95 °C for 2 min, and then 45 cycles of 95 °C for 10 s and 60 °C for 1 min.

### 3.4. Subcellular Localization of OsbHLH079

To investigate the subcellular localization of OsbHLH079, the *35S::YFP-OsbHLH079* construct was prepared. The full-length coding sequence of *OsbHLH079* was amplified with gene-specific primers (Table S1), and fused with *YFP* in the pEarleyGate 104 (pEG104) vector through LR reactions using Gateway LR Clonase II Enzyme Mix (Invitrogen, USA). The resultant construct, *35S::YFP-OsbHLH079*, and the *35S::YFP* construct were introduced into onion epidermal cells using a DNA particle delivery system (Biolistic PDS-1000/He; Bio-Rad, Hercules, CA, USA), respectively. The transformed onion epidermal cells were incubated on Murashige and Skoog phytoagar medium (pH 5.7) under dark conditions at 25 °C for 18 h, and then onion nuclei were stained with 300 nM 4′,6-diamidino-2-phenylindole (DAPI; Invitrogen, USA) in phosphate-buffered saline for 5 min. YFP and DAPI fluorescence were observed using a confocal laser scanning microscope (SP8X, Leica, Germany) with excitation wavelengths of 458 and 405 nm and emission wavelengths of 514 and 488 nm for YFP and DAPI, respectively.

### 3.5. Transactivation Activity Assay

Transactivation activity assay was performed as previously described with some modifications [91]. The full-length coding sequence of *OsbHLH079* was amplified by PCR and fused with the yeast GAL4 activation domain in the pGADT7 vector (Biosciences Clontech, Palo Alto, CA, USA), or with the yeast GAL4 DNA binding domain in the pGBKT7 vector (Biosciences Clontech, Palo Alto, CA, USA), respectively. Then, the yeast strain AH109 was co-transformed with a pair of plasmids, and plated on each medium, as shown in Figure 4c. The yeast β-galactosidase liquid assay was carried out according to the Yeast Protocols Handbook (Clontech) using chlorophenol red-β-D-galactopyranoside (CPRG, Roche Biochemical) as the substrate.

### 3.6. Scanning Electron Microscopy

Scanning electron microscopy was conducted as previously described with some modifications [92]. The lamina joints of flag leaves of WT and *osbhlh079-D* at heading stage grown under NLD conditions in the paddy field were excised and sectioned longitudinally as previously described [56]. The samples were fixed with modified Karnovsky’s fixative (2% paraformaldehyde, 2% glutaraldehyde, and 50 mM sodium cacodylate buffer, pH 7.2) at 4 °C for 24 h, and washed with 50 mM sodium cacodylate buffer (pH 7.2) three times at 4 °C for 10 min each. Next, the samples were post-fixed at 4 °C for 2 h with 1% osmium tetroxide in 50 mM sodium cacodylate buffer (pH 7.2), and washed twice with distilled water at room temperature, followed by dehydration with a gradient series of ethanol. After dehydration, the samples were processed as follows: dried in liquid CO_2_ using a critical point dryer (EM CPD300, Leica, Germany), and coated with platinum using a sputter coater (EM ACE200, Leica, Austria). The processed samples were observed by scanning electron microscope (AURIGA, Carl Zeiss, Germany).

### 3.7. BR-Induced Lamina Joint Inclination Assay

The BR-induced lamina joint inclination assay was performed as previously described with some modifications [93]. Sterilized seeds of WT and the *osbhlh079-D* mutant were grown on Murashige and Skoog (MS) medium in an artificial growth chamber at 30 °C under dark conditions for 10 days. Then, the 2-cm lamina joint segments of WT and *osbhlh079-D* were excised, and incubated on distilled water containing various concentrations of 24-epibrassinolide (BL), an active form of brassinosteroid (Sigma), at 30 °C in darkness for 48 h. The angle between lamina and sheath was measured using ImageJ software [94].

### 3.8. Gene Information

Sequence data from this article can be found in the National Center for Biotechnology Information (NCBI): *OsbHLH079*, Os02g0705500; *UBQ5*, Os01g0328400; *OsCDC6*, Os01g0856000; *OsMCM3*, Os05g0476200; *OsE2F1*, Os02g0537500; *OsCYCA3;1*, Os03g0607600; *OsEXPA3*, Os05g0276500; *OsEXPA4*, Os05g0477600; *OsEXPA5*, Os02g0744200; *OsEXPA6*, Os03g0336400; *OsEXPA7*, Os03g0822000; *OsXTH2*, Os11g0539200; *OsXTH28*, Os03g0239000; *D2*, Os01g0197100; *D11*, Os04g0469800; *BRD1*, Os03g0602300; *OsBRI1*, Os01g0718300; *OsBAK1*, Os08g0174700; *OsBSK3*, Os04g0684200; *GSK2*, Os05g0207500; *BU1*, Os06g0226500; *OsBZR1*, Os07g0580500; *ILI1*, Os04g0641700; *DLT*, Os06g0127800; *GS2*, Os02g0701300; *GS3*, Os03g0407400; *GS5*, Os05g0158500; *GS6*, Os06g0127800; *GW2*, Os02g0244100; *GW6a*, Os06g0650300; *GLW7*, Os07g0505200; *GW8*, Os08g0531600; *TGW6*, Os06g0623700; *GL7*, Os07g0603300; *PGL1*, Os03g0171300; *PGL2*, Os02g0747900; *qGL3*, Os03g0646900; *RGA1*, Os05g0333200; *RGB1*, Os03g0669200; *SRS5*, Os11g0247300; *TH1*, Os02g0811000; *BG1*, Os03g0175800; *DEP1*, Os09g0441900; *OsMAPK6*, Os06g0154500.

## 4. Conclusions

We found that OsbHLH079 increases leaf angle and grain length in rice. Rice overexpressing *OsbHLH079* (*osbhlh079-D* and *35S::OsbHLH079*) showed wider leaf angle and longer grain length, and RNA-mediated knockdown lines of *OsbHLH079* (*35S::RNAi-OsbHLH079*) exhibited narrower leaf angle and shorter grain length compared to those of WT. Our data also revealed that OsbHLH079 enhances BR signaling by modulating the expression levels of BR signaling-related genes (*OsBZR1, ILI1*, and *DLT*) which lead to increases in leaf angle and grain length. This study opens up the possibility to improve grain yield per unit area in rice by controlling plant architecture and seed shape.

## Supplementary Materials

Supplementary materials can be found at https://www.mdpi.com/1422-0067/21/6/2090/s1.

## Abbreviations

bHLH	basic Helix-Loop-Helix
BL	24-epibrassinolide
BR	Brassinosteroid
DAPI	4′,6-diamidino-2-phenylindole
GL	Grain length
GS	Grain size
GW	Grain width
LD	Long day
NLD	Natural long day
QTL	Quantitative trait loci
SD	Short day
WT	Wild-type
