# Supplementary material for: The Rice Basic Helix–Loop–Helix 79 (OsbHLH079) Determines Leaf Angle and Grain Shape
Source: Int J Mol Sci. 2020 Mar 18;21(6):2090. doi: 10.3390/ijms21062090 (PMC7139501; doi:10.3390/ijms21062090)
Supplement: Supplementary file 1 [file ijms-21-02090-s001.zip › Supplemental Figures-OsbHLH079.docx]

**Supplementary Figures** – Seo et al.


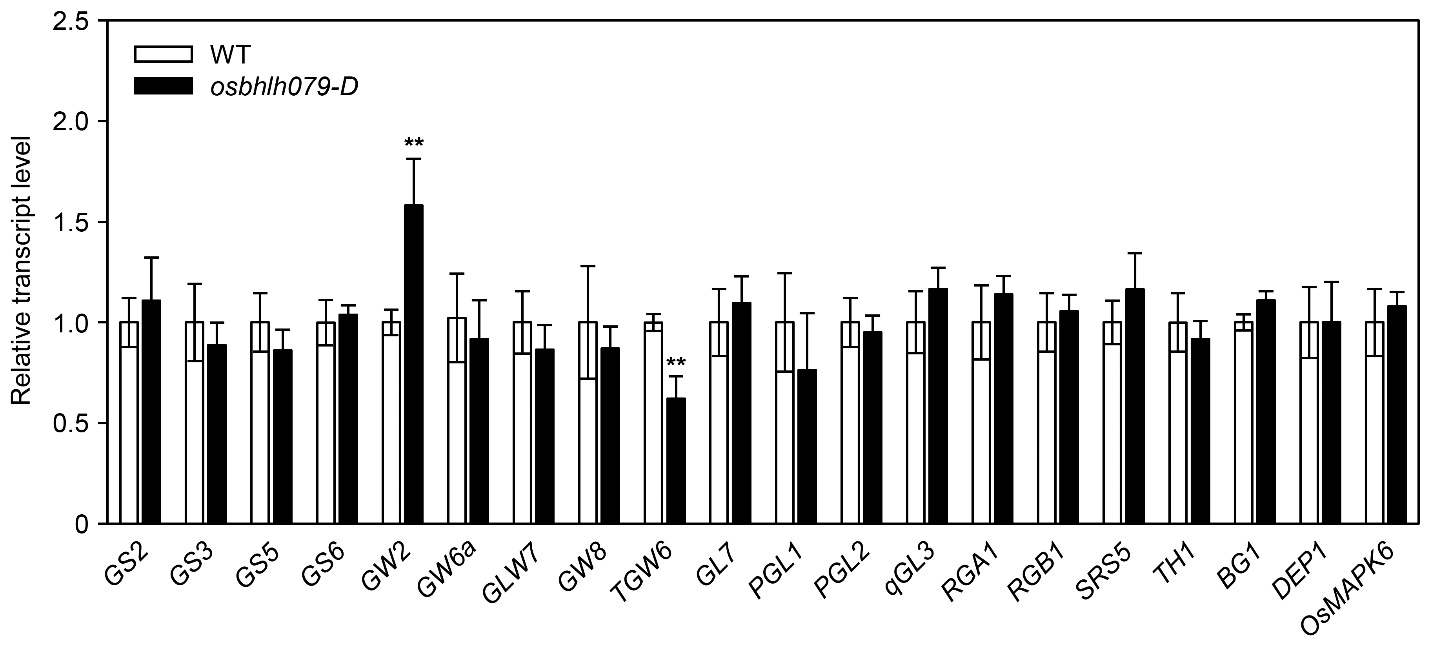


**Figure S1.** Expression patterns of grain size-related genes in the osbhlh079-D mutant compared to those in WT.

Total RNA was extracted from the panicle of WT and *osbhlh079-D* at the heading stage grown under natural long day (NLD) conditions in the paddy field. The transcript level of each gene was determined by RT-qPCR analysis and normalized to *OsUBQ5*. Means and standard deviations were obtained from three biological replicates. Significant differences between means were analyzed using Student’s *t*-test (** *P* < 0.01, Student’s *t*-test). These experiments were repeated twice with similar results.


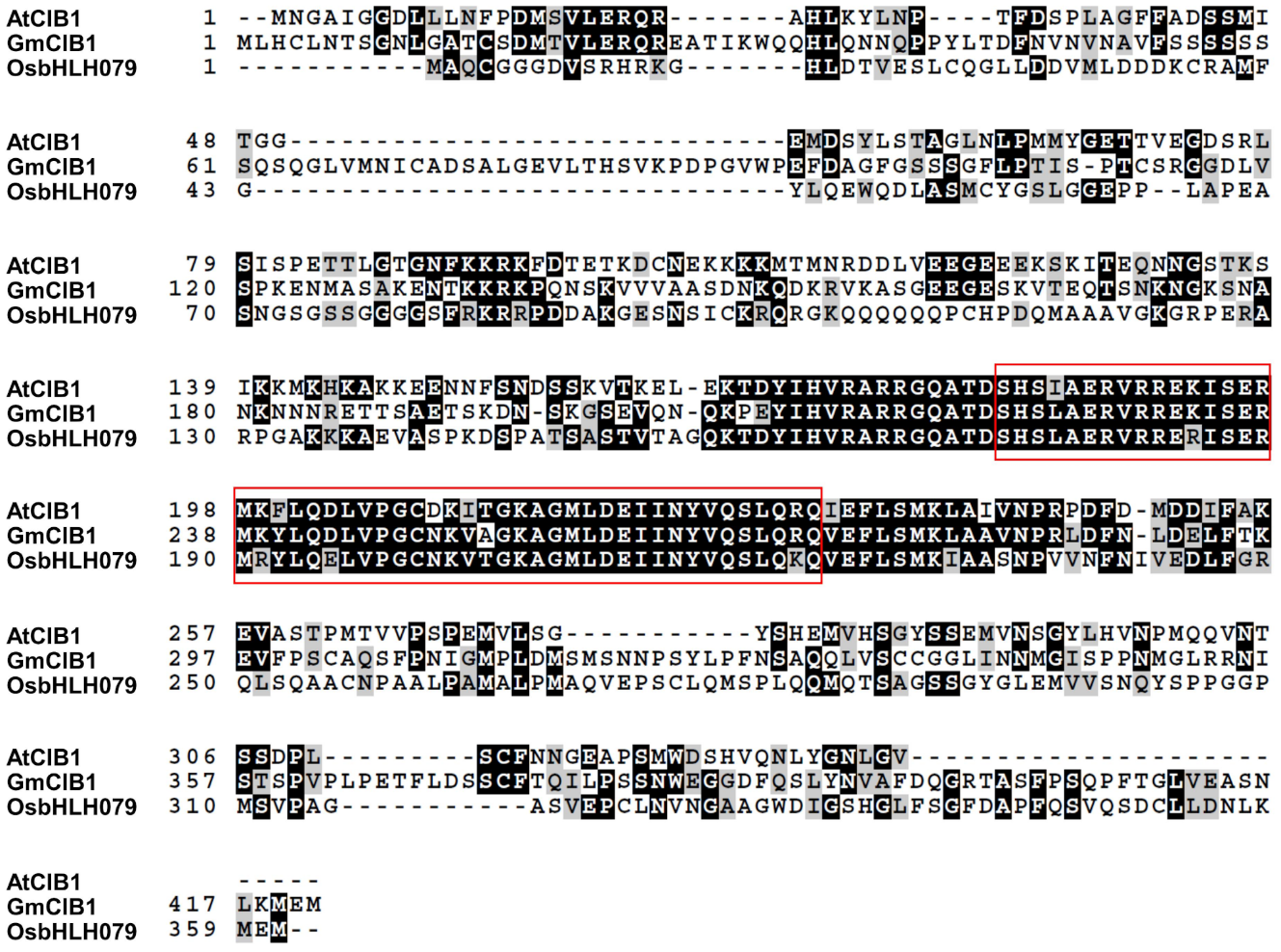


**Figure S2.** Amino acid sequence alignment of AtCIB1, GmCIB1, and OsbHLH079.

The amino acid sequences of AtCIB1, GmCIB1, and OsbHLH079 were obtained from NCBI (https://www.ncbi.nlm.nih.gov/). These amino acid sequences were aligned by ClustalW (https://embnet.vital-it.ch/software/ClustalW.html), and the graphical presentation was generated by BoxShade (https://embnet.vital-it.ch/software/BOX_form.html). Identical amino acids are shaded in black, and similar amino acids are shaded in grey. The basic Helix-Loop-Helix domains are indicated by the red box. OsbHLH079 has 41, and 36% sequence similarity to AtCIB1, and GmCIB1, respectively. AtCIB1 (*Arabidopsis thaliana* CIB1, At4g34530); GmCIB1 (*Glycine max* CIB1, Glyma11g12450).


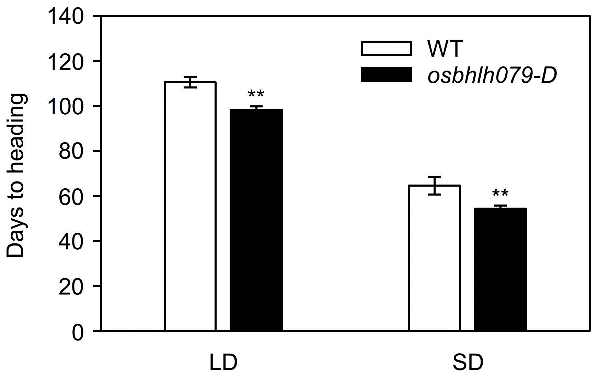


**Figure S3.** Days to heading (DTH) of WT and the osbhlh079-D mutant grown under LD (14.5 h light/day) or short day (SD) (10 h light/day) conditions with 60% relative humidity in an artificial growth chamber.

The light source was light-emitting diodes producing mixed red, blue, and white light, and the photon flux density was around 500 µmol m^-2^ s^-1^. Values are shown as means of more than ten biological replicates. Error bars indicate standard deviations. Significant differences between means were analyzed using Student’s *t*-test (** *P* < 0.01). This experiment was repeated twice with similar results.
